# Long-term archives reveal shifting extinction selectivity in China's postglacial mammal fauna

**DOI:** 10.1098/rspb.2017.1979

**Published:** 2017-11-22

**Authors:** Samuel T. Turvey, Jennifer J. Crees, Zhipeng Li, Jon Bielby, Jing Yuan

**Affiliations:** 1 Institute of Zoology, Zoological Society of London, Regent's Park, London NW1 4RY, UK; 2 Laboratory of Zooarchaeology, Center of Archaeological Science, Institute of Archaeology, Chinese Academy of Social Sciences, 27 Wangfujing Street, Beijing 100710, People's Republic of China; 3 North of England Zoological Society, Chester Zoo, Upton-by-Chester CH2 1LH, UK

**Keywords:** China, extinction filter, historical archives, Holocene, range loss

## Abstract

Ecosystems have been modified by human activities for millennia, and insights about ecology and extinction risk based only on recent data are likely to be both incomplete and biased. We synthesize multiple long-term archives (over 250 archaeological and palaeontological sites dating from the early Holocene to the Ming Dynasty and over 4400 historical records) to reconstruct the spatio-temporal dynamics of Holocene–modern range change across China, a megadiverse country experiencing extensive current-day biodiversity loss, for 34 mammal species over three successive postglacial time intervals. Our combined zooarchaeological, palaeontological, historical and current-day datasets reveal that both phylogenetic and spatial patterns of extinction selectivity have varied through time in China, probably in response both to cumulative anthropogenic impacts (an ‘extinction filter’ associated with vulnerable species and accessible landscapes being affected earlier by human activities) and also to quantitative and qualitative changes in regional pressures. China has experienced few postglacial global species-level mammal extinctions, and most species retain over 50% of their maximum estimated Holocene range despite millennia of increasing regional human pressures, suggesting that the potential still exists for successful species conservation and ecosystem restoration. Data from long-term archives also demonstrate that herbivores have experienced more historical extinctions in China, and carnivores have until recently displayed greater resilience. Accurate assessment of patterns of biodiversity loss and the likely predictive power of current-day correlates of faunal vulnerability and resilience is dependent upon novel perspectives provided by long-term archives.

## 1. Introduction

Humans have been a dominant driver of patterns in species diversity, distribution and extinction throughout recent millennia [1,2]. Integrating historical archives into macroecological research and environmental management might therefore provide novel insights on past ecosystem structure and human-mediated faunal turnover that are unavailable from short-term studies [3]. In particular, identifying biological and environmental factors that can predispose species to be vulnerable or resilient to extinction has been a major area of research during the development of predictive conservation science [4–6]. In recent years, palaeoecological research has generated substantial insights into the dynamics and ecosystem effects of biodiversity loss through geological ‘deep time’ and into the Late Quaternary [7,8]. However, correlates of extinction risk are typically studied in modern-day systems, which have experienced an ‘extinction filter’ and have already lost biodiversity that was more vulnerable to past human pressures, so that insights from such studies are therefore potentially both incomplete and biased [9]. Without a comparative assessment of long-term correlates of extinction risk, it is also impossible to determine whether risk factors remain constant through time, and therefore whether assessment of current-day species threat status is informative for predicting future extinction risk [10]. Despite their importance, however, multi-decadal or longer datasets are used in relatively few studies of extinction ecology [3], and most assessments of past species extinction risk have had to be conducted at coarse species- or country-level resolutions rather than at population-level or higher spatial resolutions, due to limited data availability and resolution [11,12].

Understanding past environmental baselines and the extent to which human activities have already disrupted biodiversity, and whether extinction selectivity is constant or changing through time, is of particular importance for eastern and southeast Asia. Asian terrestrial ecosystems are now experiencing extreme anthropogenic pressure, and contain the world's highest numbers of threatened vertebrate and plant species [13,14], and assessing the predictive power of correlates of vulnerability or resilience to regional human activities is an urgent conservation concern. This region also has a long history of human occupation [15], and has experienced increasing human overpopulation, resource overexploitation and habitat modification, with these pressures having escalated in intensity throughout much of the postglacial Holocene epoch [16–18]. The Holocene was a climatically stable interval relative to the rest of the Late Quaternary, and few if any Holocene vertebrate extinctions, global or regional, can be interpreted as non-anthropogenically mediated [2]. Asian ecosystems therefore have the potential to represent important study systems for investigating long-term human impacts on biodiversity, and employing restricted time windows for ecological analysis of Asian faunas could have particularly significant implications for understanding regional extinction dynamics and vulnerability.

Reconstructing past human-caused faunal turnover across much of southeast Asia remains hindered by limited availability of long-term archives [19]. However, China—a huge (approx. 9.6 million km^2^), ‘megadiverse’ country that contains over 10% of the world's extant mammal species and covers a diverse range of habitat types, including boreal and tropical forest, grasslands and deserts [20]—possesses a rich Late Quaternary palaeontological and zooarchaeological record containing abundant mammal material [17,19], with the potential to provide important insights into the changing historical status of regional biodiversity. These data have rarely been synthesized or investigated within a quantitative analytical framework [21]. However, they provide a unique resource for understanding extinction selectivity and faunal responses to human activities in a global conservation hotspot, and historical patterns across China's huge geographical area and megadiverse fauna have wider implications for understanding human-caused extinction dynamics through time. Here, we use a new georeferenced database of Holocene archaeological and palaeontological sites on mainland China from which wild mammals identifiable to species level have been recorded, and a further new database of historical Chinese mammal locality records, to investigate species responses to human impacts through time across a regional mammal fauna. We demonstrate how past environmental baselines provided by long-term faunal archives can provide novel and essential insights into the patterns, magnitude and drivers of biodiversity change, and can inform the use of current-day data for assessing future risk.

## 2. Material and methods

### (a) Data collection

We collected mammal locality data from mainland China (i.e. excluding Hainan and Taiwan) for three time periods: ‘modern’ (post-AD 2000), ‘historical’ (AD 1900–AD 2000) and ‘Holocene’ (11 700 BP–AD 1900). We only used two pre-modern time bins (rather than further subdivision) for three reasons: there was a lack of data across all species at a consistently more detailed temporal resolution; many Holocene archaeological sites span multiple temporal horizons, with mammal material not consistently reported from specific levels; and we used pre-twentieth century temporal boundaries to help identify wild versus domestic *Equus* and *Bubalus* species (e.g. all *Equus* records before the Late Shang can be interpreted as wild, whereas later records were conservatively interpreted as either domestic or impossible to distinguish from domestic on available data; electronic supplementary material, text S1), so we could not reconstruct ranges for these species across more subdivided pre-modern time bins. Mammals were selected as our focal group as they are the only wild animals that are well represented in Chinese Holocene sites, and they have received considerable attention in previous extinction risk studies, as their current global threat status is well understood [13,22] and large-scale macroecological and ecogeographic datasets are available for these taxa [4–6,11,12,23].

We obtained zooarchaeological and palaeontological records of skeletal remains of non-domesticated and non-commensal mammals identified to species level from the published and grey literature, and from unpublished collection data in the Institute of Archaeology, Chinese Academy of Social Sciences (Beijing), the Institute of Vertebrate Palaeontology and Palaeoanthropology, Chinese Academy of Sciences (Beijing), the Shanghai Museum of Science and Technology, the Three Gorges Museum (Chongqing) and the Shaanxi Institute of Archaeology (Xi'an) (electronic supplementary material, table S1). We also included the dataset of Holocene records of *Elaphurus davidianus* available in [24].

Most Holocene collections reported from China are now unavailable for study, few associated dates/ages represent direct radiometric dates on wild mammal specimens, and most site reports lack additional information with which to otherwise assess data quality [25,26]. We therefore had to follow original reported species identifications and site dates/cultures, and were unable to audit taxonomic or temporal data quality in a systematic manner, in contrast to some studies of Quaternary biodiversity turnover [27,28]. However, we excluded alleged Holocene palaeontological sites that are now reinterpreted as probably Late Pleistocene in age [25], and updated and standardized species taxonomy following Smith & Xie [20] and recent revisions (electronic supplementary material, text S1). We combined the ranges of (i) all Chinese *Naemorhedus* species and (ii) both Holocene Chinese rhinoceros species (*Dicerorhinus sumatrensis*, *Rhinoceros sondaicus*), and treated each grouping as a single species range for each time period, to accommodate uncertainty in species-level identification in many records of these widely recorded taxa (electronic supplementary material, text S1). We interpret all Holocene non-domesticated mammal records as representing individuals from wild populations that occurred in the vicinity of archaeological/fossil sites where they were reported (electronic supplementary material, text S1).

A minimum of six locality points is required to construct two range polygons, and therefore to assess whether species data represent single continuous polygons or fragmented distributions (see below). Absolute minimum sample sizes for generating accurate species distribution models have been proposed as between either 3 and 13 or 14 and 25, depending on type of dataset, with higher limits required for widespread species, and lower limits within these ranges still potentially flawed by statistical artefacts [29]. We therefore chose to analyse the subset of species recorded from 10 or more sites in the Holocene dataset, to allow for further robustness in sample size of locality data to build species maps but without discarding too many species from analysis. The number of reported Holocene localities on mainland China for these species varied between 10 and 111 (electronic supplementary material, table S2). Only seven species were recorded from six to nine Holocene sites (i.e. above minimum map-building threshold, but excluded from analysis). Their exclusion is supported by uncertainty over taxonomic validity (*Muntiacus gigas*), and/or increased likelihood that skeletal remains could be misidentified due to morphological similarity with related species (*Gazella subgutturosa*, *Procapra gutturosa*) [30]. These species include representatives of several mammal orders (Artiodactyla, Carnivora, Primates), and span a range of body sizes and ecologies, indicating that exclusion from further analysis is unlikely to bias our results.

We obtained most of our historical records from the compendium of Chinese mammal localities in [31], which contains data from published and unpublished Chinese sources dating from 1930 onwards, and further data on Chinese mammals from the Russian literature dating back to 1888. We supplemented this list with additional locality records [32–41], and from the entire run of the *China Journal of Science and Arts* (35 volumes, 1923–1941). Historical records were typically reported at the county level; we excluded data if they referred to larger and/or more vaguely described geographical regions (e.g. ‘central and southern areas of Jiangsu’), or if they were reported by a Western author using an idiosyncratic early transliteration system (i.e. not Wade–Giles or pinyin transliteration) and could not be matched to known modern localities. Historical data generally refer to wild mammal observations that were approximately contemporaneous with publication date of each reference, or date from a few years beforehand (although we note that some records for *Equus ferus* [36–40] refer to nineteenth century locality records, but with the assumption that the species was likely to have persisted in these regions into the twentieth century); we therefore interpret historical locality records as representing an approximate baseline for geographical distributions of wild mammal populations at the beginning of the twentieth century. For the 34 wild mammal species recorded from 10 or more sites in our Holocene dataset, two species (*Bubalus mephistopheles* and *Elaphurus davidianus*) had no twentieth-century Chinese records. The number of historical localities for other species varied between 5 and 249 (electronic supplementary material, table S2).

We used IUCN range maps as modern mammal ranges. These were downloaded from the IUCN website as vector polygon shapefiles (http://www.iucnredlist.org/technical-documents/spatial-data) and converted to cylindrical equal area projection in ArcMAP [42]. We removed parts of IUCN ranges specifically noted as regions where species were now extinct (e.g. for *Ursus arctos* and *U. thibetanus*), and included them within historical ranges. We then clipped all range maps to a map of China.

### (b) Species maps

We assigned all historical and Holocene locality points a geographical coordinate (latitude–longitude) by searching for the records in a georeferencing facility (primarily using iTouch, http://itouchmap.com/latlong.html), and then checked coordinate locations to ensure they corresponded with original Holocene or historical mapped localities (e.g. in [31]), and/or verified locations using a third reference. We built up comparative historical and Holocene ranges for each species using current-day ranges as baselines onto which locality records from older time periods were also incorporated (i.e. historical ranges combined modern and historical data; Holocene ranges also included older zooarchaeological and palaeontological data), on the assumption that species ranges are unlikely to have experienced marked natural expansions or shifts beyond the early Holocene after modern postglacial climatic and environmental conditions became established. This enabled us to reconstruct past species' range polygons (http://www.iucnredlist.org/technical-documents/red-list-training/iucnspatialresources). This method allows for reasonable comparison of relative changes in distribution between species and time periods for the same geographical area despite underlying unevenness in distribution of data (http://www.iucnredlist.org/technical-documents/red-list-training/iucnspatialresources) [43], and has been used to reconstruct species distributions using presence-only Quaternary and older fossil data [44,45].

We used IUCN guidelines for species mapping (http://www.iucnredlist.org/technical-documents/red-list-training/iucnspatialresources) to build up historical and Holocene locality points onto modern range polygons. We connected each locality point by a straight line to its two nearest features: either two other points outside the base polygon, or one point and one polygon, whichever was nearest. In the absence of two such features, we used the Chinese border rather than linking to an alternative internal feature. Almost no species in our dataset are endemic to China, but instead still occur in neighbouring countries, so connecting locality points to the border in this way is intended to capture wider extralimital distributions and reduce undersampling bias near the border. However, Holocene species locality data from elsewhere in Asia are almost completely lacking, hindering our understanding of past species distributions outside China, so using a perpendicular line from a locality point to the border makes the fewest assumptions about species’ past extralimital ranges. If IUCN ranges were fragmented into more than one polygon, we connected locality points to the nearest polygon. We otherwise assumed that ranges were continuous unless they included known topographical barriers/unsuitable habitat (e.g. Tibetan Plateau, Gobi Desert). If polygons were contained within larger ones, they were dissolved. Once we had connected all points and features, we merged polygons within each temporal layer. We then converted each layer to cylindrical equal area projection and calculated the area in square kilometres. We then calculated proportion of range lost for each species between Holocene–modern, Holocene–historical and historical–modern intervals.

Whilst some other studies (e.g. [46]) have investigated range change with historical point-locality data using an alternative area of occupancy (AOO) approach (http://www.iucnredlist.org/technical-documents/red-list-training/iucnspatialresources) [43], this method is not feasible for this study, as our baseline current-day ranges are polygons which cannot be compared directly with past point-locality data in a straightforward manner. We also sought to avoid methods such as AOO that rely heavily on the actual number and distribution of individual data, as differences in spatial patterning and quantity of past locality records available for different species reflect pre- and post-excavation biases as well as underlying ecological variation in species distributions (e.g. variation in zooarchaeological species distribution records can reflect complex variation in factors including past settlement patterns and faunal exploitation by prehistoric communities, and more recent archaeological search effort [47]).

We also reconstructed separate modern, historical and Holocene spatial patterns of mammal species richness across China. For each interval, we layered and merged all species maps. We overlaid a 100 × 100 km grid cell, and calculated number of species in each grid cell. We then calculated proportion of species lost for each grid cell between Holocene–modern, Holocene–historical and historical–modern intervals.

### (c) Statistical analysis

We used the proportion of each Holocene species range lost by the start of the twentieth century, and then further lost between the start of the twentieth century and the present, to investigate whether any biological traits affected species' susceptibility to range loss and whether susceptibility varied over time. We chose body mass and trophic level as predictors, as these are known to act as proxies for many other life-history traits [48] and have been identified as key intrinsic biological parameters associated with increased extinction vulnerability that could have driven extinction filters [11]; there were insufficient species in our sample (*n* = 34) to investigate a wider range of potential variables within a statistical framework. We also tested for an interaction between body mass and trophic level, to investigate specifically whether any signal from either predictor is driven by elevated past vulnerability of large-bodied herbivores, a pattern seen in studies of modern-day mammal extinction risk [5]. We obtained life-history data from the PanTHERIA database [23], with trophic level defined as three categories: 1 = herbivore, 2 = omnivore, 3 = carnivore, following Jones *et al.* [23]. Where direct species data were unavailable in this reference, we obtained alternative data from Smith & Xie [20] or from closely related species (*Bubalus bubalis* data for *B. mephistopheles*; *Capricornis sumatraensis* for *C. milneedwardsi*; *Equus caballus* for *E. ferus*; electronic supplementary material, text S1). We modelled proportion of range lost against body mass, trophic level and the interaction of these terms. For this species-level analysis, we used a phylogenetic generalized least-squares (PGLS) model approach to account for non-independence of species due to shared ancestry, implemented using the R package ‘caper’ [49]. We used Akaike's information criterion corrected for small sample size (AICc) to compare models, and used ΔAICc to rank them relative to the top-ranked model (model with lowest AICc). We considered all models with ΔAICc values below 2 as well supported [50]. We did not investigate variable importance via model averaging because of the small number of variables under consideration. We assessed structural goodness of fit using adjusted *r*^2^ values from the outputs of the PGLS function.

Next, we investigated environmental characteristics associated with variation in regional species losses between successive time periods. The response variable was proportion of species lost per grid cell before and after AD 1900, to control for geographical variation in former Holocene regional species richness; this variable was logit transformed [51]. We tested for the potential effect of Human Footprint Index (HFI), a composite index of relative human influence (associated with likelihood of anthropogenic exploitation, conflict, habitat loss and resource competition) derived from current human population density, land use and infrastructure [52]. We recognize that HFI data represent current-day conditions, and so might be less relevant for investigating pre-modern patterns of biodiversity turnover; however, comparable data are unavailable for pre-modern periods (especially as a single composite measure spanning multiple Holocene time points, to be comparable with our Holocene mammal dataset). We also tested for potential effects of other environmental variables that have been associated with mammal population decline. The most commonly supported variables, which we included here, are: elevation (extinction vulnerability might be associated with habitat breadth and ecological adaptability, and/or elevational variation in anthropogenic activity), annual precipitation and annual temperature (extinction vulnerability might be associated with variation in productivity and resource availability regulated by these predictors), and actual evapotranspiration and potential evapotranspiration (extinction vulnerability might be associated with variation in joint or potential availability of energy and water, as measured respectively by these indices) [48,53]. We did not model all of the combinations of these variables or their interactions, as it is important to maintain clear biological hypotheses about which combinations might be important. We therefore only modelled 10 combinations to investigate different hypotheses associated with specific effects of climatic, anthropogenic and physical factors:

— All six variables previously identified as important predictors of extinction risk might be important in predicting mammal loss (model a).
— Changing climatic factors are important extinction drivers, so physical factors (elevation, HFI) were successively excluded from analysis (models b–c).
— Actual and potential evapotranspiration are closely correlated, with similar expected relationships to extinction risk, so we excluded the former from analysis (model d).
— Annual precipitation and mean annual temperature are closely correlated and again have similar expected effects on extinction risk, so we included all variables except mean temperature and the previously excluded actual evapotranspiration to have a dataset with reduced multicollinearity (model e).
— To test additive effects of human activities and climatic changes, two of the largest extinction drivers, we included only HFI and the reduced climate dataset (model f).
— Effects of climate change and extreme weather can be most extreme at high elevations, so we included only elevation and the reduced climate dataset (model g).
— High-altitude species can be sensitive to extinction processes [54], so we modelled effects of elevation only (model h).
— Human activities are among the most important extinction drivers, so we modelled effects of HFI only (model i).
— To investigate effects of non-collinear climatic variables while excluding physical factors, we modelled effects of annual precipitation and potential evapotranspiration (model j).

We overlaid maps of predictor variables on a map of China, with data aggregated to a 100 × 100 km grid cell level. To control for known spatial variation in Holocene and historical sampling, we only analysed cells containing Holocene records when investigating pre-twentieth century regional species loss, and only analysed cells containing historical records when investigating twentieth century to current-day loss; including cells lacking pre-modern records would probably underestimate regional declines, as the absence of species records for these cells might reflect incomplete sampling rather than the true absence. We modelled how proportion of species lost changed as a function of different predictors using generalized linear models, specifying a binomial error structure. We again compared and ranked model performance using AIC and determined support for each model using ΔAIC, considering models with ΔAICc below 2 as well supported. We assessed model goodness of fit using the percentage deviance explained.

## 3. Results

**Figure 1.**
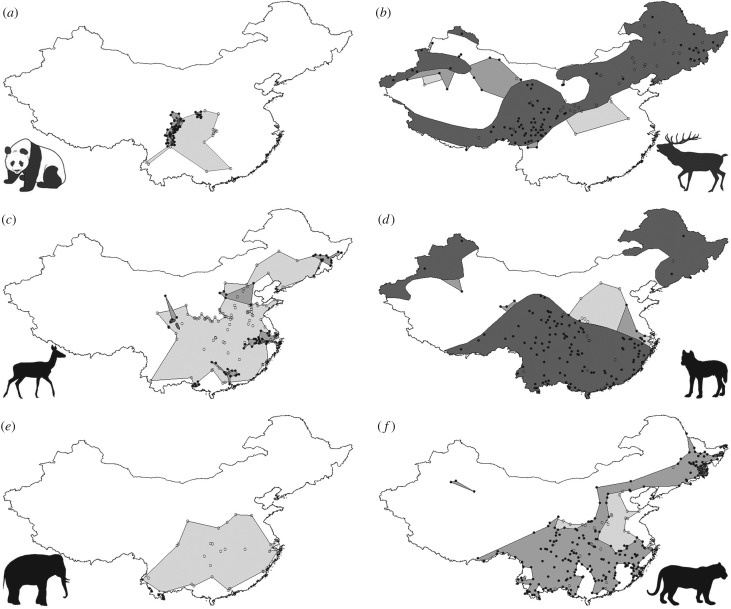
Composite range maps for six Chinese mammals, showing current-day (dark grey), twentieth-century (medium grey) and Holocene (light grey) ranges, reconstructed using historical records (filled circles) and zooarchaeological and palaeontological records (open circles). (*a*) Giant panda *Ailuropoda melanoleuca*; (*b*) red deer *Cervus elaphus*; (*c*) sika deer *Cervus nippon*; (*d*) dhole *Cuon alpinus*; (*e*) Asian elephant *Elephas maximus*; (*f*) tiger *Panthera tigris*.

Our Holocene database contains 253 Chinese archaeological and palaeontological sites with identified wild mammal species, dating from the early Holocene (approx. 11 000 BP) to the Ming Dynasty (fourteenth to seventeenth century AD), and distributed across 20 of China's 21 mainland provinces, all five provincial-level autonomous regions and three of China's four provincial-level municipalities (electronic supplementary material, figure S1 and table S1). Thirty-four wild mammal species are recorded from 10 or more sites, including representatives of Artiodactyla, Carnivora, Perissodactyla, Primates, Proboscidea and Rodentia, and comprising a broad range of biological and ecological attributes, including a body mass range of approximately 0.25–3300 kg (table 1). For these 34 species, we compiled over 4400 historical locality records from the early twentieth century onwards (electronic supplementary material, figure S1 and tables S2–S3), derived current-day geographical ranges and built up comparative twentieth century and Holocene ranges (figure 1; electronic supplementary material, figure S2), and used this series of range maps across three successive postglacial time intervals as the basis for high-resolution analysis of species responses to human impacts through time.

**Table 1.** Holocene, twentieth-century and current-day ranges for 34 Chinese mammals, including body masses and percentage of Holocene range remaining in later intervals.

species	body mass (kg)	Holocene range (km^2^)	twentieth-century range (km^2^)	current-day range (km^2^)
*Ailuropoda melanoleuca*	118.00	749 840	88 817 (11.8%)	16 285 (2.2%)
*Arctonyx collaris*	8.17	3 676 360	3 676 360 (100%)	3 657 922 (99.5%)
*Bubalus mephistopheles*	929.50	1 527 357	0 (0%)	0 (0%)
*Canis lupus*	31.76	8 721 246	8 721 246 (100%)	7 524 437 (86.3%)
*Capreolus pygargus*	41.37	4 157 171	3 820 650 (91.9%)	3 817 317 (91.8%)
*Capricornis milneedwardsii*	110.94	2 104 840	2 103 325 (99.9%)	1 999 527 (95.0%)
*Cervus elaphus*	240.87	4 816 709	4 449 413 (92.4%)	3 972 736 (82.5%)
*Cervus nippon*	53.00	2 919 625	274 792 (9.4%)	27 520 (0.9%)
*Cuon alpinus*	15.80	5 676 234	5 128 031 (90.3%)	4 956 351 (87.3%)
*Elaphurus davidianus*	165.99	963 240	0 (0%)	0 (0%)
*Elephas maximus*	3269.80	2 072 355	5461 (0.3%)	4211 (0.2%)
*Eospalax fontanierii*	0.26	1 231 580	1 213 187 (98.5%)	1 200 262 (97.5%)
*Equus ferus*	403.60	1 500 557	191 966 (12.8%)	0 (0%)
*Hydropotes inermis*	12.76	1 744 491	546 152 (31.3%)	145 161 (8.3%)
*Hystrix brachyura*	8.00	2 433 237	2 417 409 (99.3%)	2 417 409 (99.3%)
*Lutra lutra*	8.87	3 892 243	3 888 360 (99.9%)	3 785 118 (97.2%)
*Macaca mulatta*	6.46	2 937 921	2 935 508 (99.9%)	2 844 952 (96.8%)
*Meles leucurus*	6.25	6 245 111	6 245 111 (100%)	6 175 069 (98.9%)
*Muntiacus reevesi*	13.50	2 217 096	2 186 594 (98.6%)	2 186 042 (98.6%)
*Muntiacus vaginalis*	17.61	1 388 769	1 043 690 (75.2%)	1 032 519 (74.3%)
*Naemorhedus* spp.	28.22	2 213 673	2 212 241 (99.9%)	2 173 200 (98.2%)
*Nyctereutes procyonoides*	4.22	4 664 834	4 574 286 (98.1%)	4 574 274 (98.1%)
*Paguma larvata*	4.30	3 084 952	3 084 952 (100%)	3 048 167 (98.8%)
*Panthera pardus*	52.40	2 981 579	2 772 337 (93.0%)	2 659 147 (89.2%)
*Panthera tigris*	161.92	3 091 975	2 631 057 (85.1%)	29 423 (1.0%)
*Prionailurus bengalensis*	2.78	4 708 612	4 707 595 (99.9%)	4 072 294 (86.5%)
*Rhinoceros* spp.	1398.08	1 903 944	23 992 (1.3%)	0 (0%)
*Rhizomys sinensis*	1.91	2 013 597	1 964 514 (97.6%)	1 963 625 (97.5%)
*Rusa unicolor*	177.52	2 617 933	1 576 197 (60.2%)	1 561 201 (59.6%)
*Sus scrofa*	84.47	6 554 098	6 539 983 (99.8%)	6 119 878 (93.4%)
*Ursus arctos*	196.29	5 317 488	3 882 979 (73.0%)	3 364 089 (63.3%)
*Ursus thibetanus*	99.71	3 152 699	3 084 106 (97.8%)	1 696 226 (53.8%)
*Viverricula indica*	2.92	2 596 039	2 596 039 (100%)	2 591 799 (99.8%)
*Vulpes vulpes*	4.82	9 327 084	9 327 084 (100%)	9 327 084 (100%)

Mammal species vary from having lost less than 1% of their original Holocene range in China (e.g. *Arctonyx collaris*, *Hystrix brachyura*, *Viverricula indica*, *Vulpes vulpes*) to having become regionally or globally extinct (e.g. *Bubalus mephistopheles*, *Elaphurus davidianus*, *Equus ferus*) (table 1). Most species (73.5%) have lost less than 50% of their Chinese range across the Holocene, although the remaining subset have all lost over 90% of their range during this interval. In total, 22.8% of combined species' original Holocene ranges have now been lost in China, with 15.0% lost before AD 1900, and 7.8% lost after AD 1900.

For explaining the proportion of initial Holocene species’ range that was lost before AD 1900, the model with the best support (lowest AICc) contains body mass alone, although the model containing both body mass and trophic level (with no interaction) is almost equally well supported, suggesting that trophic level is also an influential predictor (table 2; electronic supplementary material, text S2). Based on these models, larger-bodied species and herbivores are both more likely to have lost relatively more geographical range before AD 1900. Both models explain almost half of total variation in past range loss (adjusted *r*^2^ = 0.430–0.469). Conversely, the strong signal of body mass for explaining range loss is lost after AD 1900. The most well-supported model now contains only trophic level (table 2), and all well-supported models explain much less of total variation in recent range loss (adjusted *r*^2^ = 0.159–0.247). It is also worth noting that if we used a ΔAICc threshold of 6 rather than 2, as suggested by [55], all four models would be considered well supported for explaining recent range loss.

**Table 2.** PGLS models investigating variation in proportion of mammal range loss in China, before AD 1900 (*a*) and after AD 1900 (*b*), and reporting maximum log-likelihood (LL), parameter count (*k*), change in Akaike's information criterion (corrected for finite sample size) relative to top-ranked model (ΔAICc) and adjusted *r*^2^.

model	LL	*k*	ΔAICc	adjusted *r*^2^
(*a*) proportion of range loss before AD 1900				
body mass	−58.960	4	0	0.430
body mass + trophic level	−61.250	2	0.499	0.469
body mass + trophic level + interaction	−58.824	6	5.979	0.435
trophic level	−65.890	2	11.714	0.219
(*b*) proportion of range loss after AD 1900				
trophic level	−50.870	2	0	0.159
body mass + trophic level + interaction	−47.301	6	1.265	0.247
body mass + trophic level	−50.680	4	2.212	0.140
body mass	−54.703	2	5.239	0.005

**Figure 2.**
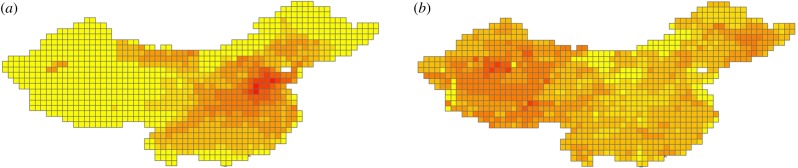
Proportion of mammal species lost per 100 × 100 km grid cell across China before AD 1900 (*a*) and after AD 1900 (*b*). Proportion of species lost increases from paler to darker squares (bins: 0, 0.1–4.0, 4.1–8.0, 8.1–12.0, 12.1–16.0, 16<).

Analysis of changing spatial patterns of mammal species richness and variation in regional losses over time across China for different intervals at a 100 × 100 km grid cell resolution (figure 2; electronic supplementary material, figure S3) shows that proportion of species lost before AD 1900 is explained by a single parsimonious model (model a) containing all six of our predictors (table 3). In this model, fewer species have been lost in grid cells with higher elevation, lower HFI, higher annual precipitation, lower annual temperature, lower actual evapotranspiration and higher potential evapotranspiration. This model explains almost half of total variation in species lost per grid cell (% deviance explained = 0.468) (electronic supplementary material, text S2). Conversely, five different models, containing different combinations of climatic, anthropogenic and physical variables (models b–f), are all well supported to explain proportion of species lost after AD 1900, with AIC values within 2 units of each other, but these models all explain only very low levels of variation (% deviance explained = 0.064–0.069; table 3).

**Table 3.** Generalized least-squares models to explain variation in proportion of species lost per 100 × 100 km grid cell across China, before AD 1900 (*a*) and after AD 1900 (*b*), and reporting maximum log-likelihood (LL), parameter count (*k*), change in Akaike's information criterion (corrected for finite sample size) relative to top-ranked model (ΔAICc) and percentage deviance explained. Abbreviations: AET, actual evapotranspiration; Elev, elevation; HFI, Human Footprint Index; PET, potential evapotranspiration; Rain, annual precipitation; Temp, annual temperature.

model	LL	*k*	ΔAICc	% deviance explained
(*a*) proportion of species lost before AD 1900				
AET + Elev + HFI + PET + Rain + Temp	−1430.126	7	0	0.468
AET + HFI + PET + Rain + Temp	−1439.070	6	15.890	0.462
Elev + HFI + PET + Rain + Temp	−1469.992	6	77.734	0.439
Elev + HFI + PET + Rain	−1498.354	5	132.458	0.418
AET + PET + Rain + Temp	−1549.076	5	233.900	0.381
HFI + PET + Rain	−1498.354	4	251.875	0.373
HFI only	−1601.421	2	332.591	0.342
Elev + PET + Rain	−1619.442	4	372.634	0.329
Elev only	−1719.010	2	567.768	0.256
PET + Rain	−1997.714	3	1127.178	0.051
(*b*) proportion of species lost after AD 1900				
AET + PET + Rain + Temp	−1533.476	5	0	0.067
HFI + PET + Rain	−1534.570	4	0.188	0.064
AET + HFI + PET + Rain + Temp	−1533.028	6	1.103	0.069
Elev + HFI + PET + Rain + Temp	−1533.101	6	1.250	0.068
Elev + HFI + PET + Rain	−1534.397	5	1.842	0.065
Elev + PET + Rain	−1535.522	4	2.091	0.062
AET + Elev + HFI + PET + Rain + Temp	−1532.787	7	2.621	0.069
PET + Rain	−1537.111	3	3.270	0.058
Elev only	−1545.201	2	17.449	0.036
HFI only	−1546.968	2	20.983	0.031

## 4. Discussion

Our findings provide new evidence for previously identified relationships between extinction risk and biological or environmental factors. Analysis of variation in species extinction risk supports the known positive relationship between extinction risk and body size, which is associated with lower population densities and intrinsic rates of increase in larger-bodied species, making them more vulnerable to anthropogenic and non-anthropogenic environmental pressures, and such species are also preferentially exploited by humans [4–6,13]. Analysis of variation in regional extinction risk supports known relationships between extinction risk and several climatic, anthropogenic and physical variables [48]; for example, populations occurring at lower elevations are known to be more vulnerable to extinction due to greater human population growth and habitat conversion in these accessible regions, and many threatened species now restricted to high-elevation refugia formerly had broader elevational distributions [21,53]. More importantly, our combined zooarchaeological, palaeontological, historical and current-day datasets reveal that both phylogenetic and spatial patterns of extinction selectivity have varied through time in China, with body mass decreasing in significance as a predictor of species extinction risk, and a marked reduction in ability of our models to explain variation in species extinction risk or regional extinction risk using any of our chosen biological or environmental variables. These novel findings demonstrate the presence of important extinction filters affecting current-day ecological data that can bias our understanding of faunal vulnerability and resilience in the absence of novel perspectives provided by long-term archives.

The changing pattern of extinction selectivity observed through time in China might reflect the cumulative impact of ongoing regional human pressures, with vulnerable species disappearing and accessible landscapes becoming modified earlier on during the Holocene, leaving a subset of ecologically resilient species and geographically remote landscapes that show reduced extinction risk. Under this model, the decreasing significance of body mass as a predictor of species extinction risk might reflect the greater level of geographical range loss shown by larger-bodied species in China before the twentieth century, with little range left to be lost for these species over the past century. Similarly, the decreasing significance of all modelled environmental factors for explaining variation in regional extinction risk might reflect the loss of many Chinese mammal populations that had become restricted by the start of the twentieth century to remnant refugia associated with specific ecological conditions (e.g. high elevations), with ‘extinction debt’ in many such landscapes that had already become too degraded to support viable populations in the long term [21].

Alternatively, shifting extinction selectivity in China's mammal fauna through time might be associated with changing regional anthropogenic pressures. Indeed, the decreasing predictive power over time shown by body mass might not be explained by a simplistic extinction filter model of near-complete pre-twentieth-century range loss in larger-bodied species, as several large-bodied mammals (e.g. *Capricornis milneedwardsii*, *Cervus elaphus*, *Rusa unicolor*, *Ursus arctos*, *U. thibetanus*) maintained wide geographical distributions across China into the twentieth century and even up to the present (table 1). Instead, whereas mammalian extinction risk in China before AD 1900 was influenced by several different environmental factors, during the twentieth century spatial extinction patterns became more homogeneous (figure 2*b*), and our predictive models lose most of their ability to explain variation in extinction risk. China therefore appears to have become a system in which the ‘field of bullets’ model of extinction selectivity is likely to apply [56], with extinction becoming effectively unpredictable in relation to life-history traits or environmental conditions, and small- and large-bodied species across different landscapes all experiencing declines. A comparable global shift in mammalian extinction selectivity across the Holocene has been interpreted as possibly indicating a change in primary driver of biodiversity loss, from overexploitation of a taxonomically restricted subset of large-bodied species to wider-scale habitat destruction [11]. Over the past century, Chinese environments have experienced a massive increase in habitat loss and natural resource exploitation, associated with the country's human population explosion and well-documented destructive environmental policies, as well as an increase in the focus and scope of harmful activities (e.g. the mid-twentieth-century ideological ‘war on nature’, when systematic politically driven campaigns led to rapid extirpation of tigers and other large carnivores that had not previously been the focus of heavy persecution) [57,58]. Geographical expansion of human pressures across China's diverse range of ecological landscapes during the twentieth century (e.g. onto the high-elevation Qinghai–Tibetan Plateau [59]) might also explain the decreasing significance of any environmental variables as good predictors of extinction risk in our analyses (figure 2*b*).

We acknowledge that it is difficult to assess the data quality for China's Holocene faunal record in a systematic manner, in terms of concerns such as robustness of species identification and site dating; such problems are by no means unique to this study and remain widespread when dealing more generally with past data [60]. Spatial non-independence, where geographically proximal locations exhibit values that are more similar than those further apart, can provide an additional source of bias by increasing sample size without contributing independent information [61]. Whereas modern-day distribution data are typically derived from thorough presence–absence surveys [62], Holocene and historical archives generally represent presence-only data, which can be affected by past and present spatial sampling biases [47]. We sought to minimize sampling biases associated with archival data by using mapping methods that are not sensitive to total number and distribution of individual data, and by analysing only grid cells containing relevant older records when investigating patterns of regional species loss through time. However, the starting point for reconstructing almost all species ranges was an IUCN polygon produced using standardized methodology and expert assessment rather than a series of discrete current-day locality points (http://www.iucnredlist.org/technical-documents/spatial-data); polygons were extended to incorporate individual historical and Holocene records, with only range extent (i.e. range edge) redefined by older point-locality records. This use of combined polygon + point-locality data prevented straightforward assessment of spatial non-independence (e.g. using distance matrices) within our species distribution datasets.

Despite these challenges, China's long-term, spatio-temporally high-resolution faunal record can still provide an extremely important new baseline for understanding the magnitude and dynamics of human-caused biodiversity loss in this conservation hotspot, and this record presents a unique perspective unavailable from modern-day datasets. Previous studies have investigated range change in a small number of mammal taxa during recent centuries or millennia using past occurrence records in China's historical gazetteer (*difangzhi*) archive, in which some mammals are identifiable to species or ‘species group’ level [21,63]. Our integrated use of multiple archives to achieve a much longer-term view of changing extinction dynamics across China's mammal fauna through the Holocene represents a further key step in the use of regional environmental records.

China's mammal fauna is recognized as being highly threatened today [20,22], but long-term Holocene archives reveal that postglacial mammalian losses to date have not yet been as severe as in some other geographical regions (e.g. the Caribbean, Australia [2,11,13]), with few global species-level extinctions and almost three-quarters of species retaining over 50% of their maximum estimated Holocene range despite millennia of increasing regional human pressures. The potential might therefore still exist for successful species conservation and ecosystem restoration. However, we recognize that this result is scale-dependent, with further local population extirpation and fragmentation likely to have occurred in many species at finer landscape levels [64], but undetected by resolution of available historical or IUCN data. Considerable attention is also paid today to conservation of large carnivores, which are interpreted as a particularly vulnerable ecological guild [65,66], but long-term data demonstrate that herbivores have experienced more historical extinctions in China and carnivores have until recently displayed greater resilience, challenging conservation prioritization based on recent data alone.

The ability of HFI to predict the spatial distribution of earlier Holocene species extinctions in China in our analysis of regional extinction risk provides the important insight that current-day anthropogenic variables can in some instances be used to hindcast past conditions. In this case, current-day high-HFI areas [52] include regions such as the North China Plain and the Yangtze River Valley, which have experienced high human population densities, cultural intensification and environmental exploitation for millennia [17,18], and also show elevated pre-twentieth-century mammal extinctions (figure 2*a*). However, our demonstration of shifting extinction patterns through time might support recognition of a modern ‘Anthropocene’ epoch, defined by qualitatively more intensive human pressures on global ecosystems during the past few decades or centuries [67]. The differences that we detect in extinction dynamics between past and present therefore have major implications for using long-term archives for environmental forecasting, and in particular for informing current-day conservation and environmental management, and for using data derived from contemporary systems to predict future patterns of extinction selectivity. Palaeontological, zooarchaeological and historical records are an invaluable resource for reconstructing pre-human environments and understanding the magnitude of human-caused biodiversity loss through time, but interpreting and extrapolating what they show requires both caution and context.

## Supplementary Material

Text S1-S2, Figs S1-S3 (combined file)

## Supplementary Material

Table S1

## Supplementary Material

Table S2

## Supplementary Material

Table S3

## Data accessibility

The datasets supporting this article are available as part of the electronic supplementary material, tables S1 and S3.
