# Supplementary material for: Long-term archives reveal shifting extinction selectivity in China's postglacial mammal fauna
Source: Proc Biol Sci. 2017 Nov 22;284(1867):20171979. doi: 10.1098/rspb.2017.1979 (PMC5719176; doi:10.1098/rspb.2017.1979)
Supplement: Text S1-S2, Figs S1-S3 (combined file) [file rspb20171979supp1.pdf]

LONG-TERM ARCHIVES REVEAL SHIFTING EXTINCTION SELECTIVITY IN CHINA'S POSTGLACIAL MAMMAL FAUNA

Samuel T. Turvey, Jennifer J. Crees, Zhipeng Li, Jon Bielby, Jing Yuan

Proceedings of the Royal Society B 10.1098/rspb.2017.1979

Supplementary Information Text S1

Taxonomic assignment of Chinese mammal records

We consider all Chinese Holocene *Hystrix* records, including material previously interpreted as the extinct species *H. kiangsenensis* [1], to represent *H. brachyura*, and all Holocene *Ailuropoda* records to represent *A. melanoleuca* rather than *A. baconi* [2,3]. Older taxonomic treatments recognised only a single species of both *Moschus* and *Naemorhedus* in China, but both genera are now interpreted as representing multiple species [4]. Currently recognised *Moschus* species are partially sympatric in China, making it impossible to identify many Holocene records of this genus to species level or to assess whether past records may represent multiple co-occurring species, so we therefore excluded *Moschus* data from further analysis; however, *Naemorhedus* species are allopatrically distributed in China and so we retained this genus in analysis, as Holocene records are likely to represent only one locally occurring representative of the genus. Holocene rhinoceros records from China include material assigned to both *Dicerorhinus sumatrensis* and *Rhinoceros sondaicus*, as well as additional material unidentified to species or genus level; we

retained all Holocene rhinoceros records in analysis as a single "species" category in order to include this important megafaunal taxon in our analyses of extinction risk, although we recognise that these composite rhino data may therefore not be strictly comparable to data for other Chinese species. We averaged life history data across all four *Naemorhedus* species and across *Dicerorhinus sumatrensis* and *Rhinoceros sondaicus* for analysis, respectively.

We interpreted all Holocene non-domesticated mammal species records as representing individuals from wild populations that occurred in the nearby vicinity of the archaeological or fossil site from which they were reported. We consider this to represent a non-controversial assumption in most cases, especially for wild species that are frequent in archaeological sites and which were therefore probably hunted locally for food. However, we note that specific body parts of some high-prestige species, notably elephant ivory, may represent trade items that could have been transported some distance by human agency. For example, it is suggested that both elephant ivory and alligator hide may have been traded between landscapes in China as early as the Neolithic [5], and this may represent a possible explanation for caches of elephant tusks reported from Chinese sites such as Jinsha [6]. We excluded all sites representing only records of modified elephant ivory to minimize potential bias in range reconstruction for this species due to inclusion of potential trade items, and we consider that the potentially uncertain archaeological status of some elephant tusks (which constitute a small proportion of our total elephant records) is unlikely to affect interpretation of our Holocene data, as ancient historical records provide independent evidence for the former wide distribution of wild elephants across China [7,8].

The issue of differentiating between wild and domestic forms of domesticated mammal taxa has constituted a major research question in Chinese zooarchaeology

[9,10], and represents an important consideration for interpreting Holocene records of these taxa. Well-established morphological protocols exist for differentiating between wolf and domestic dog, and between wild boar and domestic pig, and so we included uncontroversial records of wild forms of both taxa in our analyses. Although many Holocene *Bos* records may represent either wild *B. primigenius* [11] or potentially other now-extinct wild species (e.g., "*B. exiguus*", reported from sites such as Cishan, Hebei [12]), it has recently been argued that *Bos* may have been domesticated in northeast China at the start of the Holocene [13]; however, other researchers consider that this apparent evidence for early cattle domestication is unlikely to be related to human management [14]. As most potentially wild records of *Bos* in China remain very poorly understood and controversial, we excluded these from analysis. Similarly, there is considerable uncertainty over the identity not only of wild versus domestic forms of sheep and goats in archaeological sites, but also of which wild *Capra* or *Ovis* species or related caprine genera were formerly present across different landscapes in China [10], and so we also excluded Holocene records of these taxa from analysis. Bactrian camels were domesticated in north-central Asia 5000-4500 BP and were in common use in China by the Bronze Age Zhou Dynasty (3100-2400 BP) [11]; whereas *Camelus* records from older sites can therefore be interpreted as wild animals, we also excluded this taxon from our analyses because very few (<10) uncontroversially wild Chinese Holocene records are available.

The first domestic horses in China are recorded from the Bronze Age Late Shang site of Yinxu (c.3050 BP), which also has wild horses present in the faunal assemblage [10]; this represents a later introduction of domesticated animals from outside China through exchange and interaction of culture and commerce. Horse remains from older, Neolithic (e.g., Yangshao culture) sites (Banpo, Dabagou, Guantaoyuan,

Miaozigou) for which detailed morphological studies have been conducted have all been shown to be wild *Equus ferus* [9]. We therefore interpreted all horse remains from sites (or layers within stratigraphically complex sites) that are older than the Late Shang, and which do not represent obvious animal sacrifices more typical of interaction with domesticates (e.g., as represented by complete skeletons buried in pits), as representing wild individuals for the purposes of analysis, as are any later specimens which have been specifically identified as *E. ferus*. However, the wild or domestic status of pre-Late Shang horse remains is still not fully understood, and we note that future research may reveal a more complex spatiotemporal pattern of horse domestication in China.

There is confusion over the first appearance of domestic *Bubalus* (i.e., the domesticated "swamp buffalo" morph of *B. arnee*, rather than the endemic wild *B. mephistopheles*, which was previously and erroneously thought to be ancestral to domestic Chinese water buffalo [15]). Domestic buffalo first appear in southeast Asia in the 2nd century BC, and the earliest evidence for domestic buffalo in China is from representations in art: rock art from the 1st-5th centuries AD from Yunnan, and sculptures cast on bronze drums or clay models from tombs, all dating from the 2nd century AD or later [9]. No wild *B. arnee* specimens have been found in the Chinese zooarchaeological record, and no securely identified *B. bubalis* remains have been reported from well-dated Neolithic or Bronze Age archaeological sites; instead, all buffalo remains from Neolithic contexts belong to *B. mephistopheles* whenever material is identifiable to species (other native Chinese *Bubalus* species appear to have become regionally extinct by the Late Pleistocene). Material from some Neolithic or Bronze Age sites has been assigned to "*Bubalus* sp." mainly due to the absence of horncores in these faunal assemblages, which are typically used to identify

buffalo species [16]. *B. bubalis* remains have been reported from Haimenkou in Jianchuan, northwest Yunnan, dating to the late part of the first millennium BC, but this date is controversial and the stratigraphy of the site may have been disturbed [17]. Therefore, in addition to specimens specifically identified as *B. mephistopheles*, we assigned all material of "*Bubalus* sp." from Neolithic sites/horizons, and from Bronze Age sites/horizons dating from older than the first millennium BC, to *B. mephistopheles* (this only includes those sites with ages not overlapping the first millennium BC, in order to be conservative and avoid potential for confusion with a possible early presence of domestic buffalo in China based on the alleged first millennium BC Haimenkou "domestic" buffalo specimen). We also interpreted the identification of the Pleistocene taxon "*B. wansjocki*" from Kangjia [18] as representing *B. mephistopheles*.

References

1. van Weers DJ (2005) A taxonomic revision of the Pleistocene *Hystrix* (Hystricidae, Rodentia) from Eurasia with notes on the evolution of the family. *Contrib Zool* 74:301-312.
2. Turvey ST, Tong H, Stuart AJ, Lister AM (2013) Holocene survival of Late Pleistocene megafauna in China: a critical review of the evidence. *Quat Sci Rev* 76:156-166.
3. Jin C, et al. (2007) The first skull of the earliest giant panda. *Proc Natl Acad Sci USA* 104:10932-10937.
4. Smith AT, Xie Y (Eds.) (2008) *A Guide to the Mammals of China* (Princeton University Press, Princeton).

5. Underhill AP (2000) An analysis of mortuary ritual at the Dawenkou site, Shandong, China. *J East Asian Archaeol* 2:93-127.
6. Zhu Z, Zhang Q, Wang F (2006) The Jinsha site: an introduction. *J East Asian Archaeol* 5:247-276.
7. Elvin M (2004) *The Retreat of the Elephants: An Environmental History of China* (Yale University Press, New Haven).
8. Wen R (2009) *The Distributions and Changes of Rare Wild Animals in China* (Chongqing Science and Technology Press, Chongqing).
9. Liu L, Chen X (2012) *The Archaeology of China: From the Late Paleolithic to the Early Bronze Age* (Cambridge University Press, Cambridge).
10. Yuan J, Han J, Blench R (2008) Livestock in ancient China: an archaeological perspective. *Past Human Migrations in East Asia: Matching Archaeology, Linguistics and Genetics*, eds. Sanchez-Mazas A, Blench R, Ross MD, Peiros I, Lin M (Routledge, Abingdon and New York), pp 84-104.
11. Madsen BD, Chen F, Gao X (Eds.) (2007) *Late Quaternary Climate Change and Human Adaptation in Arid China* (Elsevier, Amsterdam).
12. Zhou B (1981) The animal remains discovered at Cishan village, Wu'an, Hebei Province. *Kaogu Xuebao* 3:339-347.
13. Zhang H, et al. (2013) Morphological and genetic evidence for early Holocene cattle management in northeastern China. *Nature Comms* 4:2755.
14. Lü P, Yuan J, Li Z (2014) Re-exploration the origination of domesticated cattle in China – discussion on the evidence of morphology and genetics about the management of cattle in the early Holocene, the northeast China. *Cultural Relics in Southern China* 3:48-59.

15. Yang DY, Liu L, Chen X, Speller CF (2008) Wild or domesticated: DNA analysis of ancient water buffalo remains from north China. *J Archaeol Sci* 35:2778-2785.

16. Liu L, Chen X, Jiang L (2004) A study of Neolithic water buffalo remains from Zhejiang, China. *Bull Indo-Pacific Prehist Assoc* 24:113-120.

17. Li X, Han R (2006) Yunnan Jianchuanxian Haimenkou yizhi chutu tongqi de jishu fenxi jiqi niandai [Technological analysis of bronzeware artefacts excavated from the Haimenkou site, Jianchuan county, Yunnan]. *Kaogu* 7:80-85.

18. Liu L (2004) *The Chinese Neolithic: Trajectories to Early States* (Cambridge University Press, Cambridge).

Supplementary Information Text S2

Models and model outputs

A. Species susceptibility to range loss, Holocene – AD 1900

Predictor = body mass

Model: pgls(Holocene_Historical_logit ~ log(AdultBodyMass), data=cdat, lambda='ML')

	**Estimate**	**Std. error**	**t**	**p**
(Intercept)	-8.349	1.467	-5.692	2.95E-06
log(AdultBodyMass)	0.690	0.138	5.011	2.08E-05

Predictor = trophic level

Model: pgls(Holocene_Historical_logit ~ factor(TrophicLevel), data=cdat, lambda='ML')

	**Estimate**	**Std. error**	**t**	**p**
(Intercept)	-0.157	0.441	-0.357	0.723
factor(TrophicLevel)2	-2.385	0.833	-2.865	0.008
factor(TrophicLevel)3	-1.933	0.794	-2.434	0.021

Predictor = body mass + trophic level

Model: pgls(Holocene_Historical_logit ~ log(AdultBodyMass) + factor(TrophicLevel), data=cdat, lambda='ML')

	**Estimate**	**Std. error**	**t**	**p**
(Intercept)	-6.514	1.673	-3.893	0.0005
log(AdultBodyMass)	0.567	0.146	3.892	0.0005
factor(TrophicLevel)2	-1.389	0.733	-1.896	0.068
factor(TrophicLevel)3	-1.040	0.694	-1.499	0.145

Predictor = body mass + trophic level + body mass*trophic level

Model: pgls(Holocene_Historical_logit ~ log(AdultBodyMass) + factor(TrophicLevel) + log(AdultBodyMass)*factor(TrophicLevel), data=cdat, lambda='ML')

	**Estimate**	**Std. error**	**t**	**p**
(Intercept)	-6.975	1.994	-3.498	0.002
log(AdultBodyMass)	0.608	0.175	3.481	0.002
factor(TrophicLevel)2	-0.066	4.294	-0.016	0.988
factor(TrophicLevel)3	0.871	4.737	0.184	0.855
log(AdultBodyMass):factor(TrophicLevel)2	-0.132	0.434	-0.305	0.763
log(AdultBodyMass):factor(TrophicLevel)3	-0.192	0.475	-0.403	0.690

B. Species susceptibility to range loss, AD 1900 – present

Predictor = body mass

Model: pgls(Historical_Modern_logit ~ log(AdultBodyMass), data=cdat, lambda='ML')

	**Estimate**	**Std. error**	**t**	**p**
(Intercept)	-2.287	1.209	-1.901	0.067
log(AdultBodyMass)	0.0448	0.113	0.397	0.694

Predictor = trophic level

Model: pgls(Historical_Modern_logit ~ factor(TrophicLevel), data=cdat, lambda='ML')

	**Estimate**	**Std. error**	**t**	**p**
(Intercept)	-1.905	0.279	-6.817	1.46E-07
factor(TrophicLevel)2	-0.726	0.528	-1.374	0.180
factor(TrophicLevel)3	0.991	0.504	1.967	0.058

Predictor = body mass + trophic level

Model: pgls(Historical_Modern_logit ~ log(AdultBodyMass) + factor(TrophicLevel), data=cdat, lambda='ML')

	Estimate	Std. error	t	p
(Intercept)	-2.651	1.302	-2.036	0.051
log(AdultBodyMass)	0.066	0.114	0.587	0.562
factor(TrophicLevel)2	-0.609	0.570	-1.068	0.294
factor(TrophicLevel)3	1.096	0.540	2.030	0.052

Predictor = body mass + trophic level + body mass*trophic level

Model: pgls(Historical_Modern_logit ~ log(AdultBodyMass) + factor(TrophicLevel) + log(AdultBodyMass)*factor(TrophicLevel), data=cdat, lambda='ML')

	Estimate	Std. error	t	p
(Intercept)	-1.040	1.406	-0.739	0.466
log(AdultBodyMass)	-0.077	0.123	-0.627	0.536
factor(TrophicLevel)2	-7.762	3.029	-2.563	0.016
factor(TrophicLevel)3	-2.387	3.341	-0.714	0.481
log(AdultBodyMass):factor(TrophicLevel)2	0.730	0.306	2.385	0.024
log(AdultBodyMass):factor(TrophicLevel)3	0.338	0.335	1.008	0.322

C. Proportion of species lost per grid cell, Holocene – AD 1900

Model a: glm(Holo_Historical ~ AET + Elev + HFI + PET + Rain + Temp, binomial)

	Estimate	Std. error	z	p
(Intercept)	3.33E+00	1.77E-01	18.762	2.00E-16
Elev	1.79E-04	4.30E-05	4.170	3.04E-05
HFI	-4.38E-02	3.76E-03	-11.630	2.00E-16
Rain	1.77E-03	1.34E-04	13.240	2.00E-16
Temp	-9.33E-03	9.58E-04	-9.737	2.00E-16
AET	-2.12E-03	2.53E-04	-8.398	2.00E-16
PET	6.07E-04	2.91E-04	2.088	0.037

Model b: glm(Holo_Historical ~ AET + HFI + PET + Rain + Temp, binomial)

	Estimate	Std. error	z	p
(Intercept)	3.533	0.173	20.449	2.00E-16
HFI	-0.050	0.004	-14.291	2.00E-16
Rain	0.002	0.001	13.543	2.00E-16
Temp	-0.011	0.001	-13.510	2.00E-16
AET	-0.002	0.001	-9.556	2.00E-16
PET	0.001	0.001	4.828	1.38E-06

Model c: glm(Holo_Historical ~ AET + PET + Rain + Temp, binomial)

	**Estimate**	**Std. error**	**z**	**p**
(Intercept)	1.723	0.109	15.770	<2e-16
Rain	0.002	0.001	17.950	<2e-16
Temp	-0.017	0.001	-25.040	<2e-16
AET	-0.004	0.001	-15.010	<2e-16
PET	0.003	0.001	12.470	<2e-16

Model d: glm(Holo_Historical ~ Elev + HFI + PET + Rain + Temp, binomial)

	**Estimate**	**Std. error**	**z**	**p**
(Intercept)	3.72E+00	1.65E-01	22.468	2.00E-16
Elev	2.63E-04	4.17E-05	6.309	2.81E-10
HFI	-4.89E-02	3.59E-03	-13.635	2.00E-16
Rain	9.89E-04	9.08E-05	10.883	2.00E-16
Temp	-6.64E-03	8.73E-04	-7.610	2.75E-14
PET	-1.03E-03	2.01E-04	-5.122	3.02E-07

Model e: glm(Holo_Historical ~ Elev + HFI + PET + Rain, binomial)

	**Estimate**	**Std. error**	**z**	**p**
(Intercept)	4.04E+00	1.66E-01	24.395	<2e-16
Elev	3.97E-04	3.79E-05	10.479	<2e-16
HFI	-5.45E-02	3.67E-03	-14.843	<2e-16
Rain	7.27E-04	8.84E-05	8.226	<2e-16
PET	-2.00E-03	1.80E-04	-11.064	<2e-16

Model f: glm(Holo_Historical ~ HFI + PET + Rain, binomial)

	**Estimate**	**Std. error**	**z**	**p**
(Intercept)	5.06E+00	1.48E-01	34.192	2.00E-16
HFI	-8.14E-02	2.94E-03	-27.715	2.00E-16
Rain	2.95E-04	7.66E-05	3.853	0.0001
PET	-1.35E-03	1.62E-04	-8.325	2.00E-16

Model g: glm(Holo_Historical ~ Elev + PET + Rain, binomial)

	**Estimate**	**Std. error**	**z**	**p**
(Intercept)	2.32E+00	1.05E-01	22.180	<2e-16
Elev	7.47E-04	3.29E-05	22.720	<2e-16
Rain	9.11E-04	8.88E-05	10.260	<2e-16
PET	-2.45E-03	1.85E-04	-13.230	<2e-16

Model h: glm(Holo_Historical ~ Elev, binomial)

	Estimate	Std. error	z	p
(Intercept)	1.19E+00	3.14E-02	37.840	<2e-16
Elev	5.99E-04	2.81E-05	21.310	<2e-16

Model i: glm(Holo_Historical ~ HFI, binomial)

	Estimate	Std. error	z	p
(Intercept)	4.069	0.092	44.290	<2e-16
HFI	-0.078	0.003	-27.730	<2e-16

Model j: glm(Holo_Historical ~ PET + Rain, binomial)

	Estimate	Std. error	z	p
(Intercept)	2.53E+00	9.82E-02	25.761	2.00E-16
Rain	-3.50E-04	6.97E-05	-5.024	5.05E-07
PET	-5.06E-04	1.53E-04	-3.308	0.0009

D. Proportion of species lost per grid cell, AD 1900 – present

Model a: glm(Hist_Modern ~ AET + Elev + HFI + PET + Rain + Temp, binomial)

	Estimate	Std. error	z	p
(Intercept)	1.70E+00	1.32E-01	12.914	< 2e-16
Elev	2.07E-05	2.98E-05	0.693	0.488
HFI	3.54E-03	3.14E-03	1.129	0.259
Rain	2.46E-04	1.19E-04	2.065	0.039
Temp	1.35E-03	7.68E-04	1.753	0.080
AET	1.56E-04	1.96E-04	0.796	0.426
PET	-9.51E-04	2.45E-04	-3.886	0.0001

Model b: glm(Hist_Modern ~ AET + HFI + PET + Rain + Temp, binomial)

	Estimate	Std. error	z	p
(Intercept)	1.710	0.131	13.010	< 2e-16
HFI	0.003	0.003	0.947	0.344
Rain	0.001	0.001	2.023	0.043
Temp	0.001	0.001	1.727	0.084
AET	0.001	0.001	0.744	0.457
PET	-0.001	0.001	-4.422	9.77E-06

Model c: glm(Hist_Modern ~ AET + PET + Rain + Temp, binomial)

	Estimate	Std. error	z	p
(Intercept)	1.796	0.096	18.778	< 2e-16
Rain	0.001	0.001	1.951	0.0511
Temp	0.001	0.001	2.593	0.010
AET	0.001	0.001	1.070	0.284
PET	-0.001	0.001	-5.425	5.79E-08

Model d: glm(Hist_Modern ~ Elev + HFI + PET + Rain + Temp, binomial)

	Estimate	Std. error	z	p
(Intercept)	1.67E+00	1.27E-01	13.218	< 2e-16
Elev	1.89E-05	2.98E-05	0.634	0.526
HFI	4.16E-03	3.04E-03	1.369	0.171
Rain	3.13E-04	8.44E-05	3.711	0.0002
Temp	1.20E-03	7.48E-04	1.603	0.109
PET	-8.62E-04	2.19E-04	-3.930	8.49E-05

Model e: glm(Hist_Modern ~ Elev + HFI + PET + Rain, binomial)

	Estimate	Std. error	z	p
(Intercept)	1.61E+00	1.20E-01	13.421	< 2e-16
Elev	-1.30E-05	2.21E-05	-0.588	0.557
HFI	4.53E-03	3.02E-03	1.501	0.133
Rain	3.36E-04	8.24E-05	4.076	4.57E-05
PET	-6.33E-04	1.61E-04	-3.922	8.79E-05

Model f: glm(Hist_Modern ~ HFI + PET + Rain, binomial)

	Estimate	Std. error	z	p
(Intercept)	1.58E+00	1.08E-01	14.592	< 2e-16
HFI	5.56E-03	2.47E-03	2.251	0.0244
Rain	3.55E-04	7.58E-05	4.685	2.81E-06
PET	-6.70E-04	1.49E-04	-4.500	6.80E-06

Model g: glm(Hist_Modern ~ Elev + PET + Rain, binomial)

	Estimate	Std. error	z	p
(Intercept)	1.73E+00	9.12E-02	18.978	< 2e-16
Elev	-3.22E-05	1.80E-05	-1.788	0.074
Rain	3.51E-04	8.17E-05	4.293	1.76E-05
PET	-6.18E-04	1.61E-04	-3.845	0.0001

Model h: glm(Hist_Modern ~ Elev, binomial)

	**Estimate**	**Std. error**	**z**	**p**
(Intercept)	1.57E+00	3.21E-02	49.038	< 2e-16
Elev	-7.65E-05	1.46E-05	-5.232	1.68E-07

Model i: glm(Hist_Modern ~ HFI, binomial)

	**Estimate**	**Std. error**	**z**	**p**
(Intercept)	1.197	0.058	20.803	< 2e-16
HFI	0.010	0.002	4.832	1.35E-06

Model j: glm(Hist_Modern ~ PET + Rain, binomial)

	**Estimate**	**Std. error**	**z**	**p**
(Intercept)	1.72E+00	9.10E-02	18.866	2.00E-16
Rain	4.34E-04	6.73E-05	6.449	1.13E-10
PET	-7.41E-04	1.46E-04	-5.087	3.64E-07

Supplementary Figures

Fig. S1. Distribution across mainland China of (A) Holocene zooarchaeological and paleontological sites from which wild mammals have been reported, and (B) historical locality data for the 34 wild mammal species recorded from ≥10 sites in the Holocene dataset.

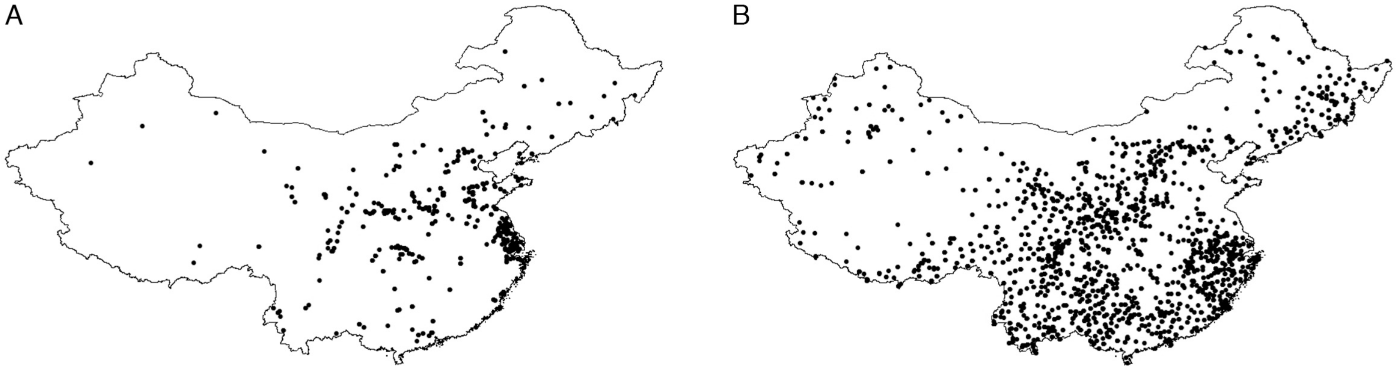
A
B

Fig. S2. Composite range maps for 34 Chinese mammals, showing current-day (IUCN; dark grey), 20th century (medium grey) and Holocene (light grey) ranges, reconstructed using historical records (filled circles) and zooarchaeological and paleontological records (open circles).

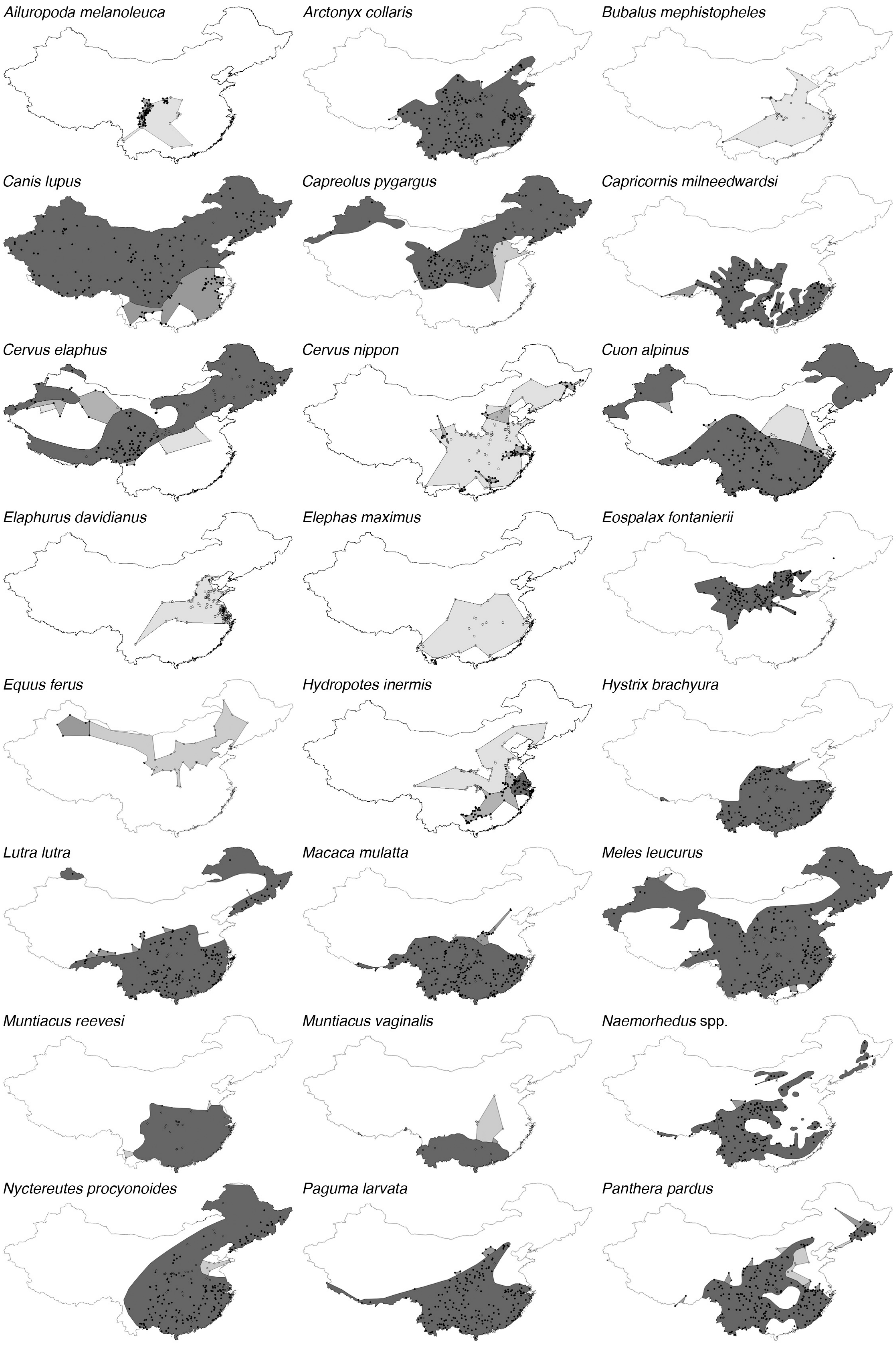
Ailuropoda melanoleuca
Arctonyx collaris
Bubalus mephistopheles
Canis lupus
Capreolus pygargus
Capricornis milneedwardsi
Cervus elaphus
Cervus nippon
Cuon alpinus
Elaphurus davidianus
Elephas maximus
Eospalax fontanierii
Equus ferus
Hydropotes inermis
Hystrix brachyura
Lutra lutra
Macaca mulatta
Meles leucurus
Muntiacus reevesi
Muntiacus vaginalis
Naemorhedus spp.
Nyctereutes procyonoides
Paguma larvata
Panthera pardus

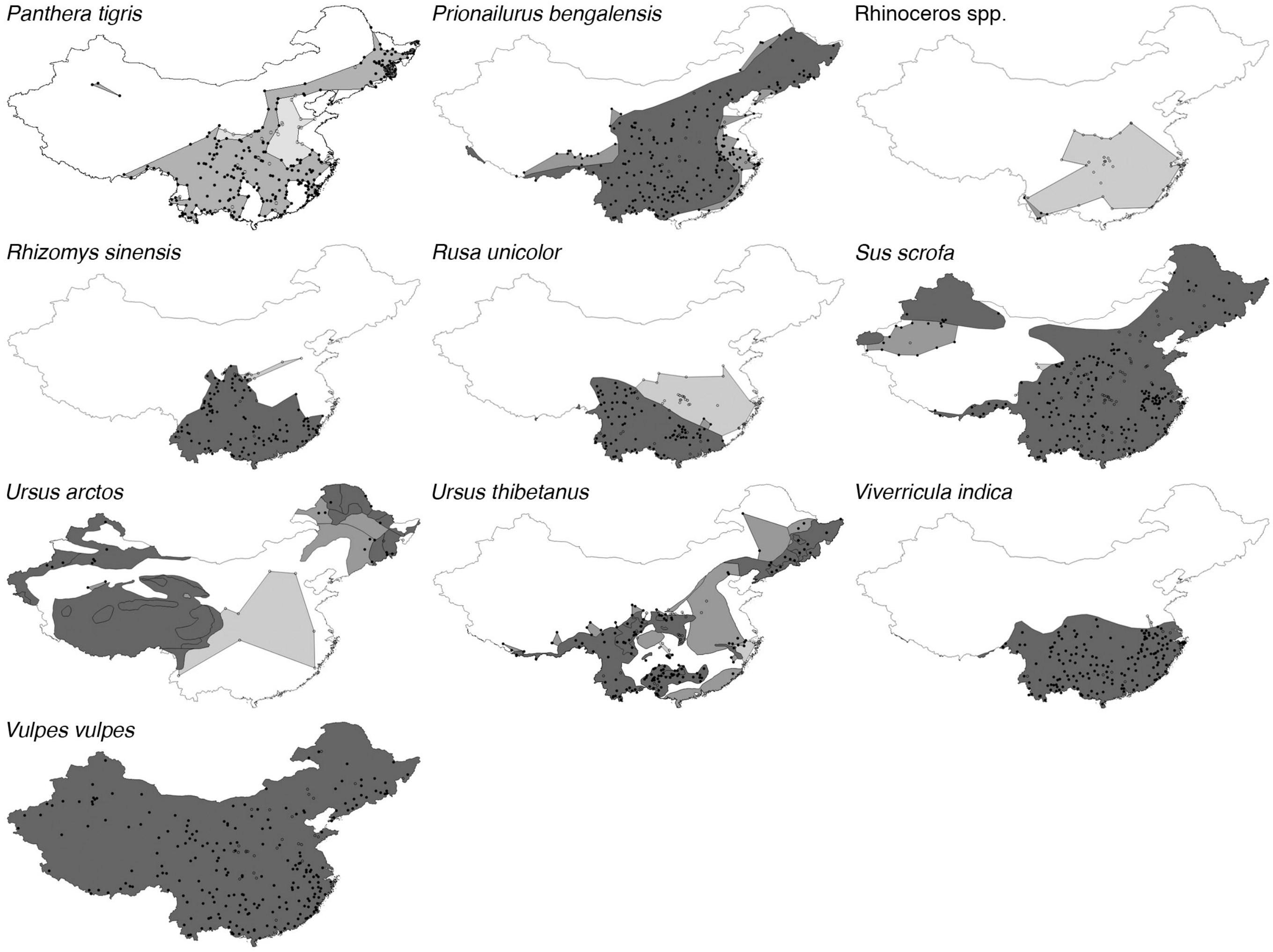
Panthera tigris
Prionailurus bengalensis
Rhinoceros spp.
Rhizomys sinensis
Rusa unicolor
Sus scrofa
Ursus arctos
Ursus thibetanus
Viverricula indica
Vulpes vulpes

Fig. S3. Total mammal species richness per 100×100 km grid-cell across China in the Holocene (A), at the start of the twentieth century (B) and today (C), based on our dataset of 34 species. Species richness increases from paler to darker squares (bins: 0-5, 6-10, 11-15, 16-20, 20<).

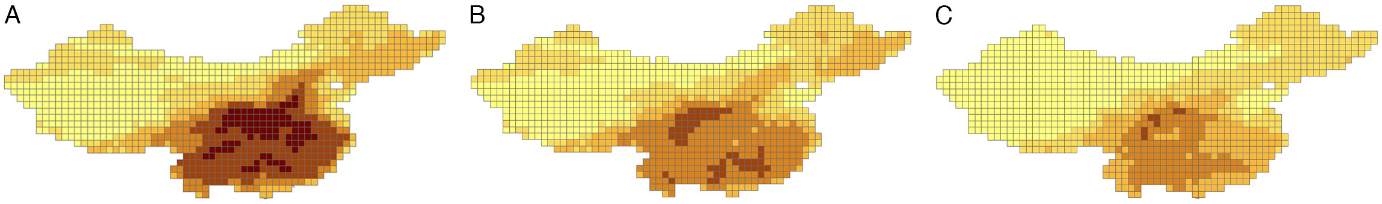

A
B
C
