# Supplementary material for: Long-term archives reveal shifting extinction selectivity in China's postglacial mammal fauna
Source: Proc Biol Sci. 2017 Nov 22;284(1867):20171979. doi: 10.1098/rspb.2017.1979 (PMC5719176; doi:10.1098/rspb.2017.1979)
Supplement: Table S2 [file rspb20171979supp3.docx]

**Table S2.** Numbers of Holocene and historical records representing separate localities available for 34 Chinese mammal species included in this study.

| **Species** | **Number of Holocene localities** | **Number of historical localities** |
| --- | --- | --- |
| *Ailuropoda melanoleuca* | 24 | 46 |
| *Arctonyx collaris* | 37 | 178 |
| *Bubalus mephistopheles* | 55 | 0 |
| *Canis lupus* | 18 | 191 |
| *Capreolus pygargus* | 51 | 119 |
| *Capricornis milneedwardsii* | 16 | 156 |
| *Cervus elaphus* | 48 | 98 |
| *Cervus nippon* | 111 | 72 |
| *Cuon alpinus* | 18 | 120 |
| *Elaphurus davidianus* | 53 | 0 |
| *Elephas maximus* | 30 | 9 |
| *Eospalax fontanierii* | 16 | 139 |
| *Equus ferus* | 40 | 12 |
| *Hydropotes inermis* | 76 | 81 |
| *Hystrix brachyura* | 40 | 118 |
| *Lutra lutra* | 13 | 194 |
| *Macaca mulatta* | 30 | 208 |
| *Meles leucurus* | 53 | 231 |
| *Muntiacus reevesi* | 25 | 143 |
| *Muntiacus vaginalis* | 12 | 85 |
| *Naemorhedus* spp. | 14 | 134 |
| *Nyctereutes procyonoides* | 52 | 160 |
| *Paguma larvata* | 10 | 200 |
| *Panthera pardus* | 25 | 222 |
| *Panthera tigris* | 42 | 236 |
| *Prionailurus bengalensis* | 14 | 249 |
| Rhinoceros spp. | 39 | 5 |
| *Rhizomys sinensis* | 19 | 112 |
| *Rusa unicolor* | 48 | 88 |
| *Sus scrofa* | 69 | 235 |
| *Ursus arctos* | 11 | 24 |
| *Ursus thibetanus* | 33 | 150 |
| *Viverricula indica* | 12 | 161 |
| *Vulpes vulpes* | 27 | 228 |
